# Real-time forecasting of infectious disease dynamics with a stochastic semi-mechanistic model

**DOI:** 10.1016/j.epidem.2016.11.003

**Published:** 2018-03

**Authors:** Sebastian Funk, Anton Camacho, Adam J. Kucharski, Rosalind M. Eggo, W. John Edmunds

**Affiliations:** Centre for the Mathematical Modelling of Infectious Diseases, London School of Hygiene & Tropical Medicine, London, United Kingdom

**Keywords:** Forecasting, Real-time modelling, Infectious disease dynamics, Outbreak

## Abstract

Real-time forecasts of infectious diseases can help public health planning, especially during outbreaks. If forecasts are generated from mechanistic models, they can be further used to target resources or to compare the impact of possible interventions. However, paremeterising such models is often difficult in real time, when information on behavioural changes, interventions and routes of transmission are not readily available. Here, we present a semi-mechanistic model of infectious disease dynamics that was used in real time during the 2013–2016 West African Ebola epidemic, and show fits to a Ebola Forecasting Challenge conducted in late 2015 with simulated data mimicking the true epidemic. We assess the performance of the model in different situations and identify strengths and shortcomings of our approach. Models such as the one presented here which combine the power of mechanistic models with the flexibility to include uncertainty about the precise outbreak dynamics may be an important tool in combating future outbreaks.

## Highlights

• A Bayesian semi-mechanistic model was applied to the Ebola Forecasting Challenge.
• Model fits to simulated data were obtained from particle Markov-chain Monte Carlo.
• Posterior samples of model parameters were used to generate forecast trajectories.
• The forecasts were assessed using subsequently released simulation points.

## 1 Introduction

Forecasting the incidence of infectious diseases is an important part of public health and intervention planning. This was especially true during the 2013–2016 West African Ebola epidemic, when the rapid expansion of the outbreak triggered an enormous national and international public health response in late summer 2014. From November 2014, the Centre for the Mathematical Modelling of Infectious Diseases (CMMID) at the London School of Hygiene & Tropical Medicine produced weekly situation reports presenting updates of publicly available epidemiological data, model fits and forecasts, and estimates of key epidemiological parameters. These reports were distributed to a wide range of public health planners, policy makers, field workers and academics in several countries by email, and were made publicly available on a dedicated web site (Center for the Mathematical Modelling of Infectious Diseases, 2015).

The forecasts in these situation reports were produced using a stochastic semi-mechanistic model of Ebola transmission (Camacho et al., 2015b). The model was mechanistic in the sense that it relied on a compartmental description of the epidemiological status of the population based on known aspects of Ebola infection such as the incubation rate or infectious period. To model transmission between individuals, however, we used a more phenomenological, stochastic approach. During an emergency such as the Ebola epidemic, it is difficult to determine the precise factors underlying disease transmission that are required to inform a fully mechanistic model. Information about the relative importance and intensity of transmission in the community, hospital or at funerals (Faye et al., 2015), about the exact extent of control measures and their impact (WHO Ebola Response Team, 2015), about behavioural changes in the community (Funk et al., 2014) as well as about the potential role of seasonality (Groseth et al., 2007) or genetic changes in the virus (Carroll et al., 2015) were not available in real-time. To capture the overall change in transmission arising from these different mechanisms, we modelled transmission between individuals using a time-varying stochastic rate.

Capturing the uncertainty in transmission in a stochastic term gives the model the flexibility to match the data in the presence of noise and uncertainty. In addition, the inferred trajectories of the transmission rate can directly be interpreted as change in the reproduction number and thus provide valuable information for decision makers, for example by indicating how far the outbreak is from being under control.

Here, we present model fits and forecasts generated as part of the Ebola Forecasting Challenge conducted in 2015 after the true epidemic had waned. The challenge was based on four scenarios of synthetic data inspired from the outbreak in Liberia outbreak, with increasing levels of noise and uncertainty (see Vespignani et al., in this issue). We used a similar model to the one used during the Ebola epidemic fitted to the simulated outbreak trajectories generated as part of the Challenge to produce forecasts of the number cases at upcoming time points. We particularly focus on methodological issues and forecasting performance. We report results on county-level data of scenario 1, and assess forecasts at time points 1, 2, 4 and 5 (time points 3 was not considered for logistical reasons). Results for the other scenarios are shown in the supplementary material.

## 2 Methods

**Fig. 1:**
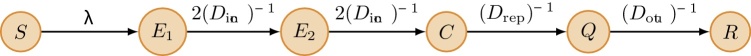
Flow between compartments of the transmission model.

Our semi-mechanistic model of Ebola dynamics is a modified Susceptible-Exposed-Infectious-Recovered (SEIR) model, accounting for delays in notification and time-varying transmission (Fig. 1). At time *t*, the force of infection experienced by susceptible individuals is *λ*_*t*_ = *β*_*t*_*I*_*t*_/*N*, where *I*_*t*_ is the overall number of infectious individuals, *N* the population size and *β*_*t*_ the time-varying transmission rate which follows a random walk (Dureau et al., 2013):

(1) $$d\log\beta_{t}=\sigma {\mathrm{dW}}_{t},$$

Here, *W*_*t*_ denotes a Wiener process (Durrett, 1984), *σ* the volatility of the transmission rate and the log-transform ensures positivity of *β*_*t*_. Modelling the time-varying transmission rate as a random walk means it is auto-correlated: the transmission rate on any day is most likely to be the same as on the previous one.

Upon infection at rate *λ*_*t*_, susceptible individuals (*S*) move from being exposed (*E*) to being infectious at a rate given by the reciprocal of the incubation period (1/*D*_inc_). We used two exposed sub-compartments in sequence to obtain an Erlang-distribution of the incubation period with shape *k* = 2 (Lloyd, 2001), and split the estimated initial number of exposed individuals evenly between these two sub-compartments. To account for delays in reporting of new cases, the infectious compartment was split into two compartments representing infectious but not yet reported (*C*) and infectious and potentially reported (*Q*) cases. The transition between *C* and *Q* occurs at a randomly varying rate with a mean equal to the reciprocal of the average reporting delay (1/*D*_rep_) and 10% overdispersion following a Gamma distribution. This stochastic variation is introduced to capture non-independence in the time until cases get reported, in order to capture situations where, for example, several members of the same family might be reported simultaneously (Camacho et al., 2015b). Lastly, infectious individuals are removed (*R*) when they recover or die from the *Q* compartment at a rate equal to the reciprocal of the difference between the infectious period and the reporting delay (*D*_out_ = *D*_inf_ − *D*_rep_). The model can be formulated as a set of stochastic differential equations which was simulated with the noise term fixed for a time step of 1 day and a Runge–Kutta method solving the remaining ordinary differential components (Milstein and Tretyakov, 2004). The only stochastic components are the trajectory of the transmission rate and the reporting noise, in contrast to the model used for the situation reports during the true Ebola epidemic, which also included demographic noise.

The observation process was modelled to operate on the weekly incidence (*Z*_*t*_), given by the number of infectious individuals entering the Q compartment. The observed incidence (${\tilde{Z}}_{t}$) was assumed to follow a normal approximation (chosen for computational efficiency) to the negative binomial distribution with reporting probability *p* and overdispersion *ϕ*:

(2) $${\tilde{Z}}_{t}∼N(pZ_{t},p(1-p)Z_{t}+p^{2}Z_{t}^{2}\phi^{2}).$$

where standard deviations smaller than 1 were rounded up to 1 to avoid the singularity at *Z*_*t*_ = 0. Note that stochastic variation here captures variability in the probability that cases get reported, whereas the stochastic variation acting on the transition from *C* to *Q* captures variability in the delay until cases can get reported.

The model thus has 8 parameters, which we either estimated from the line list of cases, took from a study on a pre-2014 outbreak of Ebola (Camacho et al., 2014), or estimated from model fits process (Table 1). Prior ranges of the transmission rate volatility and reporting overdispersion were established in preliminary runs and chosen to be able to sufficiently capture sudden changes in cases without allowing a degree of variation that would render the algorithm unstable.

**Table 1:** Parameters used in the model and their values/ranges.

Parameter	Value or prior range	Description	Source
*D*_inc_	variable	Mean delay from infection to symptoms	line list (where available)
	6 days		(Camacho et al., 2014)
*D*_rep_	1 week	Mean delay from symptom onset to notification	assumption
*D*_inf_	variable	Mean delay from symptom onset to outcome	line list (where available)
	7.8 days		(Camacho et al., 2014)
*p*	0.7	Proportion of cases reported	(Camacho et al., 2014)
*σ*	U(0,0.5)	Volatility of the transmission rate	Fitted
*ϕ*	U(0,0.5)	Overdispersion in reporting	Fitted
*E*^★^	U(0,5)	Initial number of exposed individuals	Fitted
$R^{★}$	U(0,5)	Initial reproduction number	Fitted

We used a Metropolis-Hastings particle Markov chain Monte-Carlo (pMCMC) algorithm to sample from the joint posterior distributions of the estimated parameters and states of the model (i.e. the trajectories). In brief, at each MCMC step, a particle filter is used to estimate the likelihood of the proposed parameter set, and to generate a sampled trajectory of the states of the model and the transmission rate *β*_*t*_ from their marginal posterior distribution (Andrieu et al., 2010).

Our forecasts were generated under a “no change” hypothesis: we assumed that the transmission rate would remain constant after the last observed data point. More precisely, we sampled 10,000 parameter sets from the posterior distribution in combination with the associated states and estimated values of *β*_*t*_ at the last observed data point, and simulated the model forward one year. The future number of reported cases was generated by applying the observation process to the forecast incidence. Predicted reported peak incidence, death counts and final sizes were calculated from the sampled forecast observation trajectories. County-level forecasts were obtained under the assumption that no transmission occurred between counties.

Model fits were generated using a fully automated algorithm applied to all the regional and national data sets as follows, implemented to facilitate convergence of the computationally intensive pMCMC sampler and to avoid long burn-in and low effective sample sizes: First, Metropolis-Hastings MCMC was run on the deterministic equivalent of the transmission model (with constant transmission rate and no multiplicative noise) to determine a reasonable starting location in parameter space. Then, the covariance matrix of the multivariate normal proposal distribution of the MCMC was adapted using the empirical variance in each parameter within accepted proposals, iterating and adapting an overall scale factor until an acceptance rate between 0.1 and 0.5 was achieved. Using the last parameter sample from this procedure in the fully stochastic model, the number of particles in the particle filter was calibrated numerically as the minimum number of particles required to obtain a variance of the likelihood estimator below 1. Starting from the covariance matrix of the proposal distribution obtained by fitting the deterministic model, the fully stochastic model was fitted using pMCMC, with the calibrated number of particles, and the proposal distribution was then again adapted using the same procedure as in the first step. This adapted proposal was used to run a final pMCMC for 10,000 iterations. The procedure was implemented using *libbi* (Murray, 2013) and the R packages *rbi* (Jacob and Funk, 2016) and *rbi.helpers* (Funk, 2016). A typical model fit took 5–20 min on an 8-core 2.5 GHz node (with 8 parallel threads). All the code used is freely available at http://github.com/sbfnk/ebola_forecasting_challenge.

## 3 Results

**Fig. 2:**
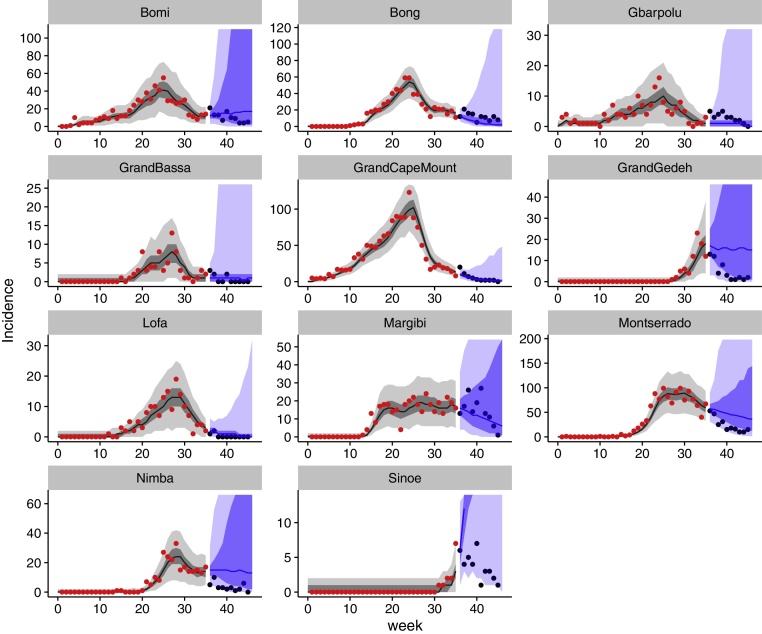
Fitted (black) and predicted (blue) incidence at time point 4 (week 35) of scenario 1. Median lines and 50% (dark) and 95% (light) credible interval ranges are shown, calculated across all trajectories at every time point. Fitted data points are shown in red, future data points (not included in the fits) in black. (For interpretation of the references to colour in this figure legend, the reader is referred to the web version of the article.)

**Fig. 3:**
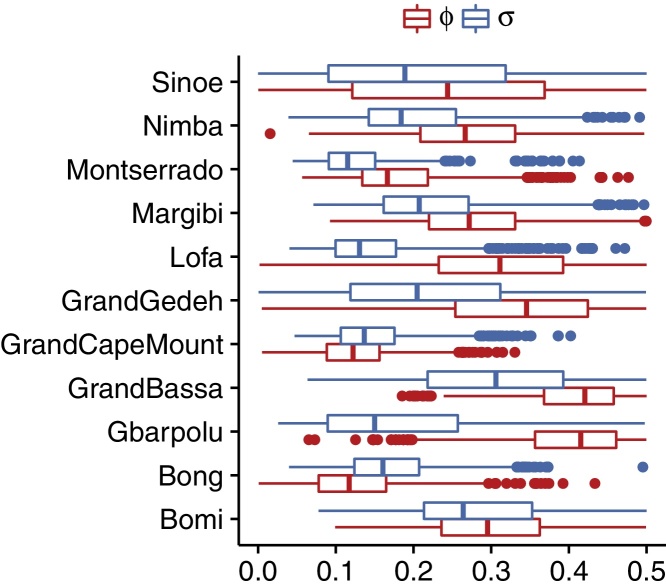
Parameter estimates of the transmission rate volatility *σ* (top, blue) and reporting overdispersion *ϕ* (bottom, red) at time point 4 (week 35) of scenario 1. Shown are the median (vertical bar), interquartile range (box), the most extreme values within 1.5 times the interquartile range (outer lines) and outliers (dots). (For interpretation of the references to colour in this figure legend, the reader is referred to the web version of the article.)

Our model was able to reproduce the observed trajectories both in scenarios of exponential increase followed by rapid decline as well as more sustained unchanged transmission (see Fig. 2 for fits to an example time point of an example scenario, others are shown in the supplementary material). Greater variability in individual trajectories was captured by greater volatility in the transmission rate (*σ*) as well as overdispersion in the observation process (*ϕ*) (Fig. 3). The two corresponding noise terms enter the model in different ways, and the estimated variability in each depends on the trajectory in question. In the model, transmission noise is correlated in time because the transmission rate is assumed to follow a random walk. In other words, the transmission rate at any time varies stochastically around the transmission rate at the previous time. Observation noise, on the other hand, is uncorrelated, and the proportion of cases reported at any time is independent of the proportion of cases reported at a previous time.

**Fig. 4:**
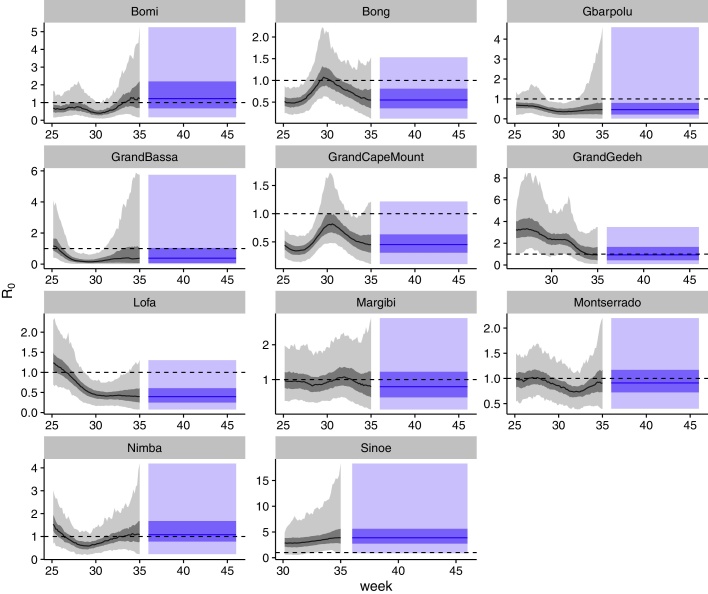
Fitted (black) and predicted (blue) trajectories of the transmission rate at time point 4 (week 35) of scenario 1, shown here rescaled with the infectious period to correspond to the reproduction number $R_{t}$. Median lines and 50% (dark) and 95% (light) credible interval ranges are shown. The horizontal dashed lines are at $R_{t}=1$. (For interpretation of the references to colour in this figure legend, the reader is referred to the web version of the article.)

Trajectories of the regional reproduction numbers ($R_{t}=\beta_{t}*D_{\inf}$ in the absence of significant depletion of susceptibles) showed a decline over the course of the epidemic in most counties, with occasional peaks and sometimes longer periods of a reproduction number close to 1, indicating the potential for sustained transmission (Fig. 4). There was no obvious pattern in the *R*_0_ trajectories, indicating that any attempt at finding a mechanistic basis for the time-varying behaviour of the transmission rate would have had to take into account differences between regions.

**Fig. 5:**
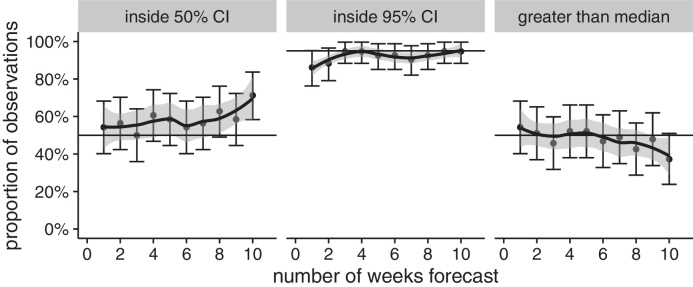
Forecasting performance, shown as proportion of data points (across regions) within the 50% credible interval (left), 95% credible interval (centre) and above the median (right) as a function of the distance in weeks predicted ahead. Shown are the mean and Bayesian 95% confidence interval using a conjugate beta prior (Gelman et al., 2013) across time points and counties of scenario 1. Trend lines for the means were obtained using locally weighted smoothing.

Retrospectively comparing the credible intervals of our district-level forecasts made at 4 with data provided later showed a decline in reliability as the forecast horizon increases (Fig. 5). The number of data points inside the 50% credible interval was slightly but consistently underestimated, with 50% and 60% of data points inside the interval, while errors were consistent with a level of 50%. This bias became stronger when we increased the forecasting horizon, with more than 70% of data points inside the 50% credible interval at 10 weeks forecast, and errors no longer consistent with a level of 50%. Further, the number of data points inside the 95% credible interval was slightly but consistently underestimated, while consistent with a level of 95%. At this level, no obvious change in performance could be observed as the forecasting horizon was expanded to 10 weeks. Lastly, our predictions were consistent with 50% of data greater than the predicted median. There was, however, a declining trend in the proportion of observations greater than the median, hinting at an overestimation of cases as the forecasting horizon increases.

## 4 Discussion

We used a stochastic semi-mechanistic model of Ebola transmission to fit the simulated trajectories in the Ebola Forecasting Challenge, and to produce forecasts that were compared to following data points. Our model is flexible enough to accommodate changes in the transmission rate that might occur due to unobserved processes. This is particularly useful in an outbreak situation where a combination of behavioural changes, potential biological changes such as viral evolution, and interventions by a variety of national and international organisations make it practically impossible to identify the key drivers of the outbreak dynamics required to inform a fully parameterised mechanistic model.

While the predictions of the model were good when nothing changes, there were divergences when predictions were made just before the epidemic peaks and the transmission rate declines (Fig. 2, Grand Gedeh), where there was resurgence or successive waves (Fig. 2, Gbarpolu), or where the epidemic was highly stochastic due to the small number of reported cases (Fig. 2, Sinoe). In the latter case, including demographic stochasticity (Keeling and Rohani, 2008) may have improved the reliability of the forecasts.

More generally, the quality of the forecasts could be seen to decrease as the forecasting horizon was expanded, with the number of data points inside the 50% credible interval increasing to more than 70%, and fewer than 40% of data points greater than the median. This would indicate that the model tended to overestimate the expected number of cases. A possible explanation for this is that all the data provided to us followed a scenario of an overall decreasing reproduction number, while our forecasts were derived assuming an assumption of no change in the transmission rate.

While the simulated trajectories presented in the Ebola Forecasting Challenge resembled the true trajectories of the West African Ebola epidemic, our model allowed for a broad range of possible scenarios including widespread epidemics where there was no clear evidence of a sustained decline. Some of our predictions may therefore appear to vastly overestimate future case numbers, but without any further information on the context of the epidemics, these scenarios could not be deemed unrealistic. Moreover, our model did not contain any mechanism for predicting increases or decreases before they occurred. In a real situation, other information could have been sourced to exclude some scenarios, or to find indicators of an impeding increase or decline. With respect to the Ebola Forecasting Challenge, using the provided situation reports may have helped yielded such information.

We chose not to change the model between forecasting time points, to provide a fair assessment of the performance of our model. However, a number of changes could have been made to improve the particular predictions requested. Our assumption of no change from the last data point meant that the forecasts were highly sensitive to fluctuations in the transmission rate at the prediction point. At that point, the model was relatively poorly informed by the data. The data at time *T* only inform the trajectory of the transmission rate up to time *T* − *D*_rep_ − *D*_inc_, that is the latest data point minus the delays because of reporting and incubation periods. After that, the transmission rate mostly follows an random walk on the logarithmic scale, which creates the potential for large variations in the transmission rate that are then propagated into the prediction. A possible improvement would have been be to average over the transmission rate over the last few time points. For the weekly forecasts we generated during the true West African Ebola epidemic, we presented both forecasts based on an average of transmission rates over the last three weeks and forecasts based on the last value.

Replacing our stochastic model for the transmission rate with a finite-variance random walk such as an Ornstein-Uhlenbeck process (Doob, 1942) would have allow us to propagate the estimated volatility instead of keeping the transmission rate fixed. Moreover, our parameter estimates including the volatility of transmission rate were informed by the entire fitted trajectories including early stages of the epidemic which may not have been relevant to later times, especially the declining phase. An approach where only the last few data points are used in the fits that inform the forecasts may have yielded more reliable forecasting results.

Because of its relatively simple mechanistic skeleton, our model is more suitable for short-term predictions of incidence than long-term predictions of final size or peak size and timing. The stochastic nature of the transmission rate allows the model to flexibly fit any trajectory and could mask underlying mis-specifications in the deterministic core. Our approach does, however, come with the important advantage of being able to test the impact of interventions or to inform vaccine trials, as has been successfully done during the West African Ebola epidemic (Camacho et al., 2015a). With this key capability, mechanistic models such as the one presented here, combined with improvements in forecasting methodology, can be expected to play a key role in informing the response to future outbreaks.

## Appendix A Supplementary data

Fits and predictions for all scenarios and time points. See the caption of Fig. 2 for details.
