# Supplementary material for: Real-time forecasting of infectious disease dynamics with a stochastic semi-mechanistic model
Source: Epidemics. 2018 Mar;22:56–61. doi: 10.1016/j.epidem.2016.11.003 (PMC5871642; doi:10.1016/j.epidem.2016.11.003)
Supplement: Supplementary file 1 — Fits and predictions for all scenarios and time points. See the caption of Fig. 2 for details. [file mmc1.docx]

**Supplemental material**

**Figures**

**Figure 1**. Temporal progression of the four simulated EVD outbreak scenarios of the Ebola Challenge. All Forecasting time points (dashed vertical lines) and new EVD cases (red line with circles) are shown.

**
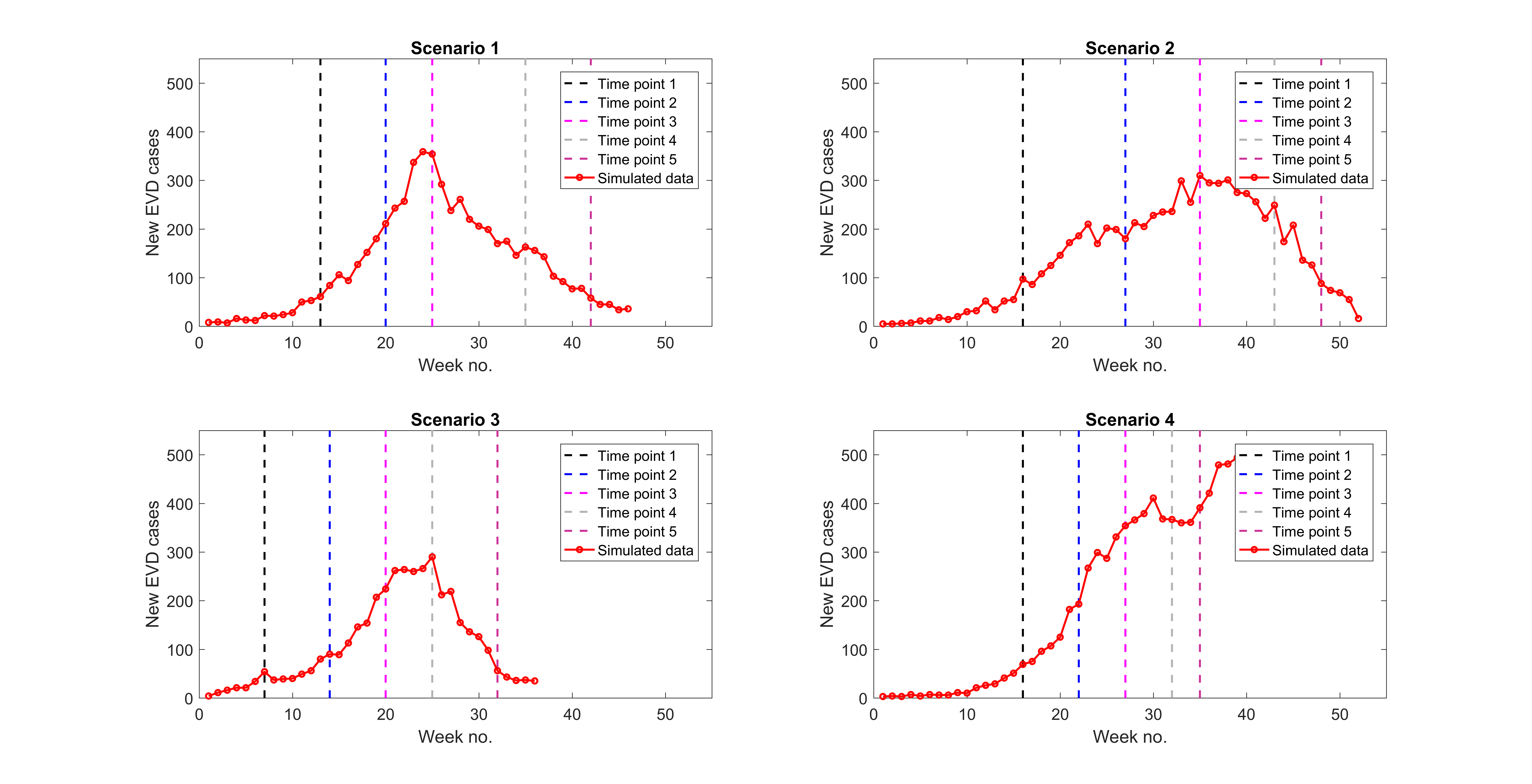
**

**Figure S2.** Distribution of generation times (blue bars) and the fitted gamma distribution (red line) for scenarios 1, 3 and 4. Note that scenario 2 did not provide any transmission tree data.

**
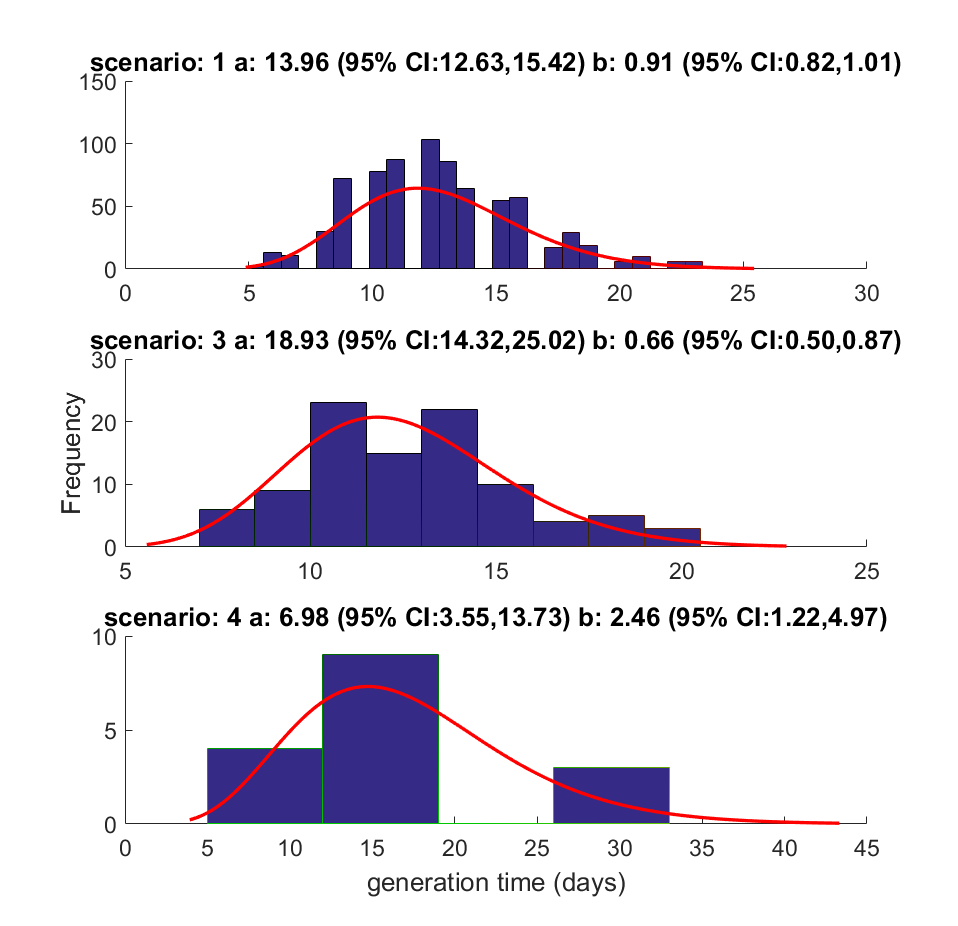
**

**Figure S3:** Means of the predicted (solid lines) epidemiological quantities and their true values (dashed lines) for the logistic growth model with method 1 (left column) and method 2 (right column).

**
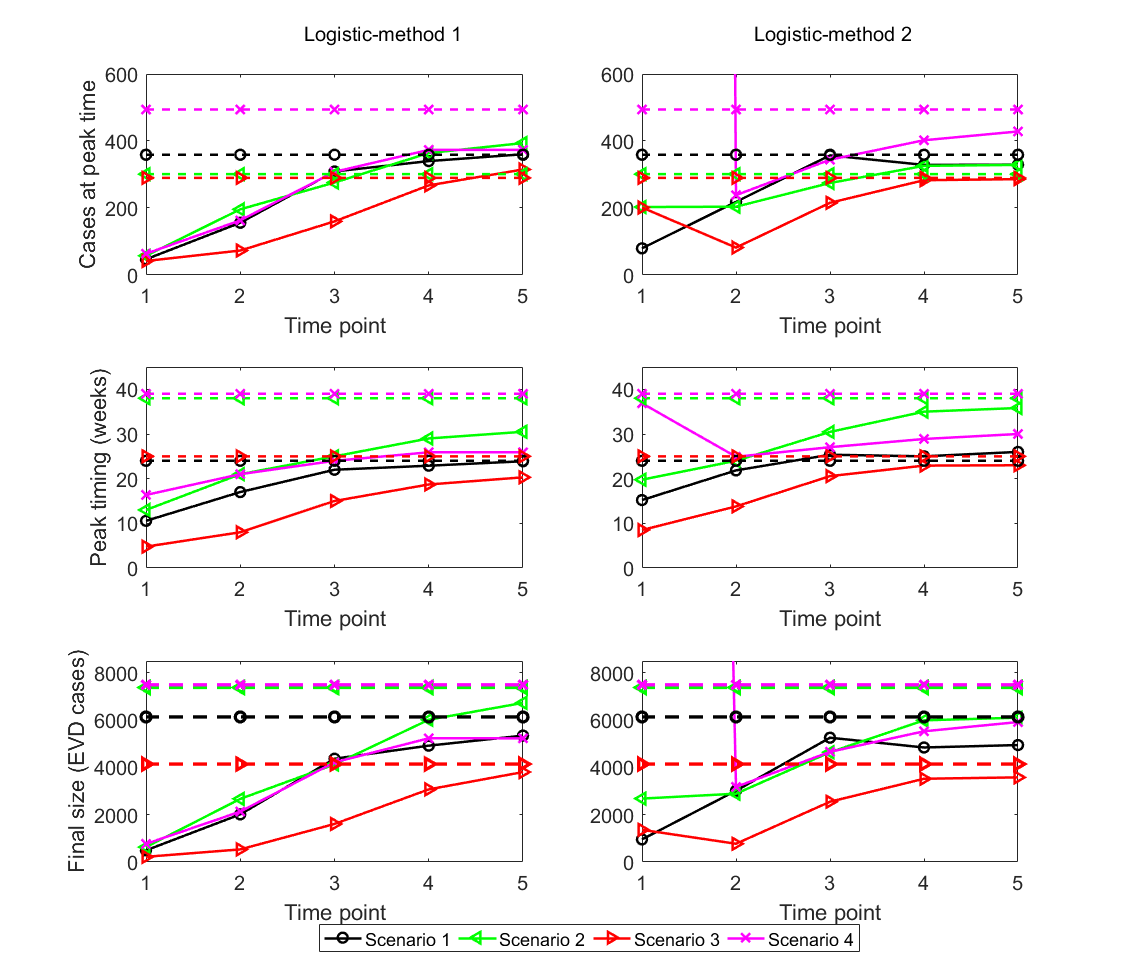
**

**Figure S4**. Means of the effective reproduction number throughout the challenge predicted by the logistic growth model using Uncertainty Method 1 (left column) and Uncertainty Method 2 (right column). For each scenario, estimations are based on an increasing number of data points that were made available during time points 1-5. Observed effective reproduction numbers (red circles) are displayed for comparison.

**
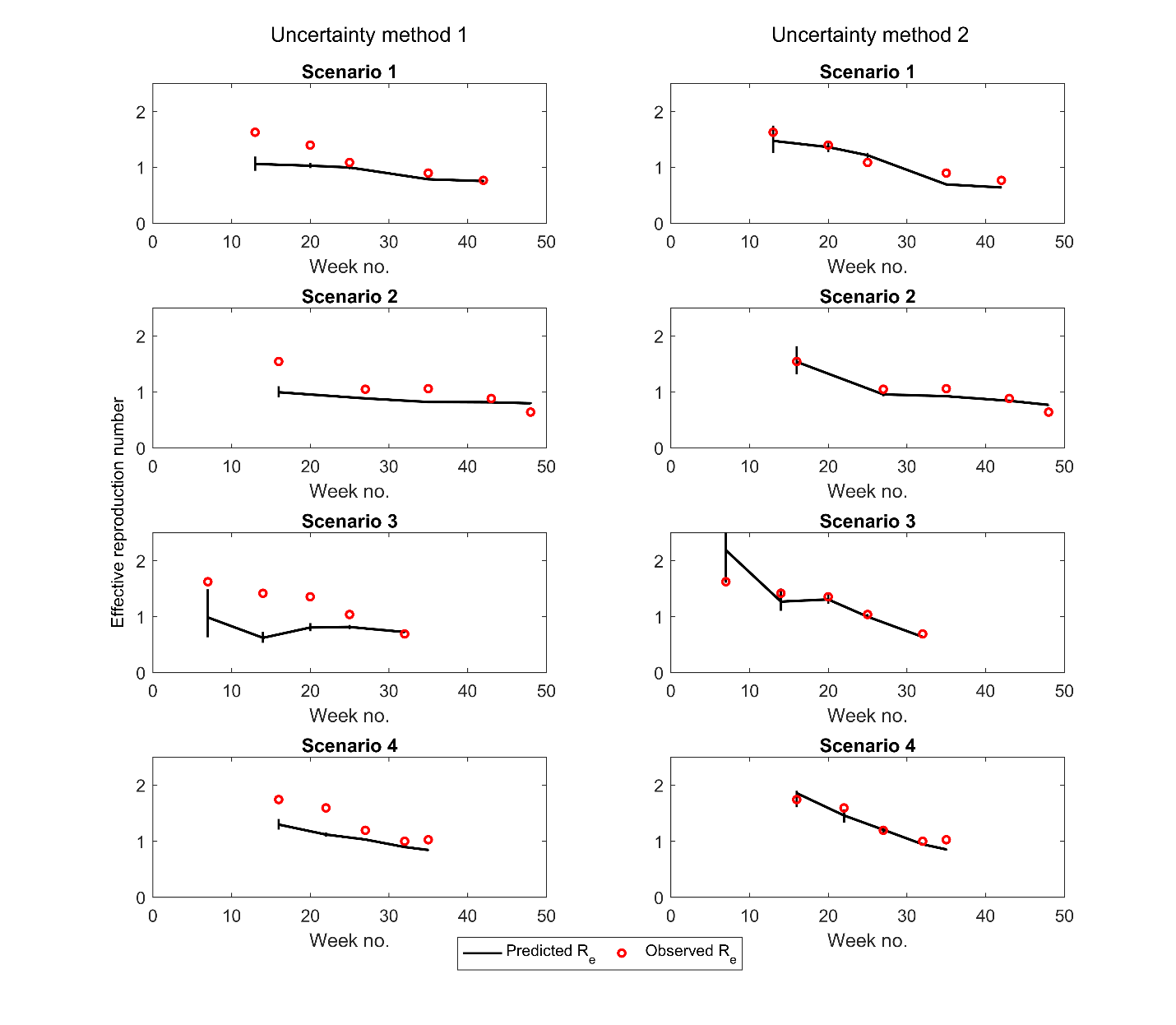
**

**Figure S5**. (Left column) Epidemic forecasts of new cases based on the logistic equation using uncertainty method 1 calibrated on an increasing amount of epidemic data: all 5 time points for scenarios 1, 2, 3 and 4, respectively. (Right column) The same epidemic forecasts seen from plotting the cumulative case counts. In both columns, the mean (solid blue line), 95% CI prediction cone (shaded blue area) for the calibrated model of 200 forecasting ensembles (grey shaded regions) are displayed along with the synthetic epidemic data (red line).

**
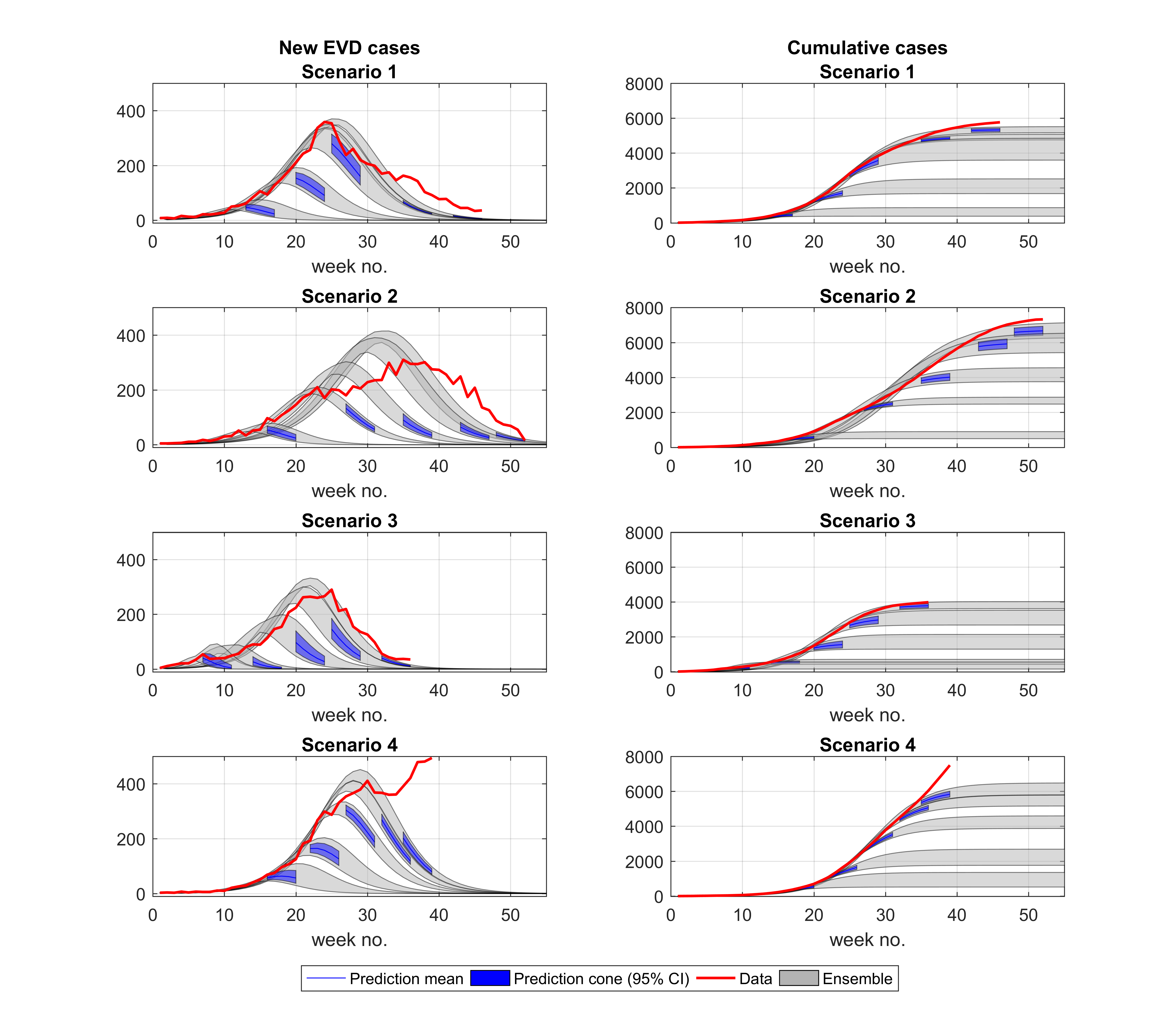
**

**Tables**

**Table S1.** Summary of mean performance statistics of the incidence targets for the logistic growth model using method 1, during the challenge. The last columns averages values across all scenarios.

|  | Scenario 1 | Scenario 2 | Scenario 3 | Scenario 4 | All Scenarios |
| --- | --- | --- | --- | --- | --- |
| R^2^ | -0.41 | -2.36 | -1.91 | -13.73 | -0.95 |
| Pearson's R | 0.72 | 0.58 | 0.55 | -0.24 | 0.7 |
| MSE | 15172.34 | 26988.96 | 20969.51 | 63362.58 | 31623.35 |
| RMSE | 123.18 | 164.28 | 144.81 | 251.72 | 177.83 |
| MAE | 99.4 | 143.79 | 124.03 | 235.55 | 150.69 |
| MAPE | 0.64 | 0.75 | 0.79 | 0.6 | 0.7 |

**Table S2**: Predicted and observed values of the effective reproduction number, using Uncertainty Method 2.

|  | GRM | Logistic | Observed R |
| --- | --- | --- | --- |
| Scenario 1 |  |  |  |
| Time point 1 | 1.464044 | 1.472521 | 1.626 |
| Time point 2 | 1.388054 | 1.360954 | 1.397 |
| Time point 3 | 1.273328 | 1.215282 | 1.086 |
| Time point 4 | 0.797766 | 0.693366 | 0.897 |
| Time point 5 | 0.793438 | 0.639503 | 0.767 |
| Scenario 2 |  |  |  |
| Time point 1 | 1.468186 | 1.536812 | 1.543 |
| Time point 2 | 1.029908 | 0.95558 | 1.048 |
| Time point 3 | 1.113256 | 0.923403 | 1.058 |
| Time point 4 | 1.052039 | 0.841311 | 0.883 |
| Time point 5 | 0.796717 | 0.768385 | 0.64 |
| Scenario 3 |  |  |  |
| Time point 1 | 1.953896 | 2.185338 | 1.622 |
| Time point 2 | 1.255024 | 1.266331 | 1.415 |
| Time point 3 | 1.367148 | 1.305004 | 1.353 |
| Time point 4 | 1.132716 | 0.996913 | 1.036 |
| Time point 5 | 0.628136 | 0.635265 | 0.688 |
| Scenario 4 |  |  |  |
| Time point 1 | 1.665946 | 1.856382 | 1.742 |
| Time point 2 | 1.462156 | 1.458677 | 1.593 |
| Time point 3 | 1.221143 | 1.203091 | 1.193 |
| Time point 4 | 1.049445 | 0.943563 | 0.998 |
| Time point 5 | 1.01137 | 0.85237 | 1.025 |
